# CROSS-CULTURAL DIFFERENCES IN RECRUITING OLDER ADULTS IN DESIGN RESEARCH ACTIVITIES: INSIGHTS FROM CHINA AND THE UK

**DOI:** 10.1093/geroni/igae098.2347

**Published:** 2024-12-31

**Authors:** Tongxin Sun, Yuanyuan Liu, Qiong Zhang, Jiabei Jiang, Xueying Xiong, Juncheng Wen, Lynne Corner, Jihong Jeung

**Affiliations:** Tsinghua University, Beijing, Beijing, China (People’s Republic); School of Mechanical Engineering & Automation, Beihang University, Beijing, Beijing, China (People’s Republic); School of Mechanical Engineering & Automation, Beijing, Beijing, China (People’s Republic); Nanjing university of the arts, Nanjing, Jiangsu, China (People’s Republic); UK’s National Innovation Centre for Ageing (NICA), Newcastle Upon Tyne, England, United Kingdom; Newcastle University, Newcastle upon Tyne, England, United Kingdom; UK’s National Innovation Centre for Ageing (NICA), Newcastle Upon Tyne, England, United Kingdom; Tsinghua University, Beijing, Beijing, China (People’s Republic)

## Abstract

Engaging older adults in the design process of products and services is essential for enhancing accessibility, usability, and the overall impact of human-centered design research focused on aging populations. Achieving successful recruitment of older adults is pivotal for their meaningful participation. Yet, the post-pandemic landscape has brought significant challenges in engaging and recruiting this demographic, underscoring the complexity of meeting their emotional, social, and cognitive needs during the recruitment phase. Furthermore, existing research predominantly concentrates on recruitment within health intervention and clinical studies, offering limited insight into effective recruitment strategies in design research across diverse socio-cultural and economic settings. Our research employed mixed-methods studies in China and the UK, incorporating surveys, interviews, and researcher immersion to investigate cross-cultural differences in recruiting older adults. We explored their attitudes, needs, and preferences, revealing the significance of treating the recruitment process as a service provided to prospective older participants. Our findings identify key socio-cultural factors—including personal identity, regional context, value systems, technology adoption, and social networks—that influence older adults’ experiences during recruitment. The reconfiguration of these factors may be required to design recruitment services and improve the participation experience. This research offers insights into cross-cultural strategies for effectively engaging and recruiting older adults in design research. By highlighting the importance of inclusivity and diversity, our work contributes to the advancement of human-centered design practices, ensuring they resonate more profoundly with aging populations.

